# Is there value beyond safety in CMR imaging of PM/AICD patients?

**DOI:** 10.1186/1532-429X-17-S1-P168

**Published:** 2015-02-03

**Authors:** Robert W Biederman, June A Yamrozik, Ronald B Williams, Geetha Rayarao, Diane V Thompson, Huma Samar, Moneal Shah, Mark Doyle

**Affiliations:** Cardiac MRI, Allegheny General Hospital, Pittsburgh, PA USA; Cardiology, VA Medical Center, Los Angeles, CA USA

## Background

Pacemaker/ AICD imaging is currently clincally performed in the MRI environment. A vigilant team consisting of Cardiologist, EP staff, and technologists with close patient monitoring and supervision has evolved this heretofore risky endeavor into a procedure with established safety if done properly. However, once safety is established, does such add valuable irrefutable information to merit that risk?

## Hypothesis

We propose that MRI in patients with a PM/AICD is crucial to confirm existing diagnosis while in many instances modifies inital diagnosis and subsequent patient management.

## Methods

A total of 100 pts were imaged on a GE CV/i Excite 12, 1.5 T (GE, WI): 17 AICD, 10 PM/AICD, 5 single PM lead (1 retained SVC AICD lead fragment), 8 REVO PM and 62 PM. A specific criteria was followed for all the pts undergoing this procedure to determine if final diagnosis provided additional information in pt care. A checklist of 3 questions were gathered and answered folowing final interpretation of the MRI.

1. Does diagnosis change?
2. Does MRI provide additional information to the existing diagnosis?
3. Does patient management change?

If 'yes' was answered to any of the above questions it was considered that MRI scan was of value to final diagnosis.

## Results

All pts completed MRI with no adverse events. The PM was interrogated pre/post MRI by EP Lab and reprogrammed under the direction of the Cardiologist. Average scan was 20±55min. Considering the 100 pts imaged: 71 (70%) were neurology cases, 5 muscoskeleton (5%) and 24(23%) were cardiac/vascular cases.

After reviewing the results from the 73 neurology cases and comparing to prior studies (CT, angio, EEG and/or myelogram), 17/71 (24%) showed the MRI not only provided additional information but changed the original diagnosis and in turn their course medical treatment. 37 (53%) provided additional information to the diagnosis. Thus, 54 pts (76%) demonstrated the MRI was of value to the final diagnosis. 17/73 (24%) pts imaged provided no further information but confirmed original diagnosis. Of 5 muscoskeletal 4(80%) provided additional information and 1(20%) changed management. The 24 cardiac cases were compared to prior studies (Cath, TEE, TTE and stress) and in 5 (21%) the MRI provided additional information to change the original diagnosis and subsequent management. Finally, 18 (75%) provided extra information while 1(4%) confirmed the original diagnosis. In essence, 96% of the cardiac population benefited by having an MRI.

## Conclusions

The use of PM/AICD imaging in MRI remains controversial but with a cautious cardiac team increased confidence in its use is found. Herein, we show that MRI procedures on carefully selected patients with pacemakers/AICD's are beneficial and substantially add valuable irrefutable information to patient diagnosis and management. We propose that not only are Pacemakers/AICD's no longer *forbidden* in the MRI environment but they can be markedly effective with life-altering and life-saving consequences.

## Funding

Internal.

